# High risk of intestinal colonization with ESBL-producing *Escherichia coli* among soldiers of military contingents in specific geographic regions

**DOI:** 10.1007/s10096-023-04684-9

**Published:** 2023-10-19

**Authors:** E. Literacka, M. Konior, R. Izdebski, D. Żabicka, M. Herda, M. Gniadkowski, K. Korzeniewski

**Affiliations:** 1 Department of Epidemiology and Clinical Microbiology, National Medicines Institute, Warsaw, Poland; 2 Department of Epidemiology and Tropical Medicine, Military Institute of Medicine - National Research Institute, Warsaw, Poland; 3 Department of Molecular Microbiology, National Medicines Institute, Warsaw, Poland

**Keywords:** *Escherichia coli*, ESBL, Carbapenemase, Intestinal carriage, Soldiers, Acquisition

## Abstract

One-hundred Polish soldiers of a contingent in Afghanistan in 2019 were screened for *Enterobacterales* resistant to newer-generation *β*-lactams at their departure and return. Seventeen percent were colonized in the gut at the departure, whereas 70% acquired carriage in Afghanistan. The commonest organisms were extended-spectrum *β*-lactamase (ESBL)-producing *Escherichia coli* (ESBL-Ec; 96.6%). All isolates were sequenced and were clonally diverse overall, even within the same sequence type, indicating that independent acquisitions mainly. ESBL-Ec were often multi-drug-resistant. Soldiers stationing in certain regions are at high risk of acquiring resistant bacteria that may cause endogenous infection, be transmitted to vulnerable individuals, and spread resistance genes.

## Introduction

Polish soldiers have been participating in international missions in different countries, often in world regions of broad dissemination of antimicrobial-resistant (AMR) pathogens, such as extended-spectrum β-lactamase (ESBL)-producing *Enterobacterales* [1–4]. Staying in such areas has been shown to be of increased risk of gut colonization with these. For example, around 75%, 49%, and 43% of Dutch travelers to Southern, Central-Eastern, and Western Asia, respectively, acquired such organisms, most often ESBL-producing *Escherichia coli* (ESBL-Ec) with the CTX-M-15 enzyme [2]. The percentage of ESBL (CTX-M-15)-Ec carriers among French soldiers stationing in Afghanistan for one year was 34.5% [5]; however, research on AMR pathogens in military contingents has been scarce so far. Various factors may specifically facilitate acquisition of pathogens in such populations, including gathering for prolonged periods, shared social areas of limited size, common sources of food and water, and/or lower sanitary standards in the field and combat conditions.

The *E. coli* intestinal carriage is a significant source of endogenous infection [3, 6–10], and diseases caused by ESBL-Ec strains are difficult to treat because of their usual multi-drug-resistance (MDR) [3, 4, 9, 11–13]. The global increase in the ESBL-Ec occurrence, raising serious clinical and epidemiological concern, has been largely due to horizontal ESBL gene transmission and/or clonal spread [3, 9, 12], the latter often associated with specific MDR clones, like *E. coli* ST131 [14]. ESBLs have been the main factor of enterobacterial resistance to expanded-spectrum β-lactams (cephalosporins), followed by acquired AmpC-like cephalosporinases and, recently, carbapenemases [15]. Our aim was to investigate the intestinal carrier status and characteristics of *Enterobacterales* resistant to newer β-lactams isolated from Polish soldiers of a contingent in Afghanistan.

## Materials and methods

From June to November 2019, 295 Polish soldiers served in Afghanistan, 100 of whom, without any symptoms of illness, were tested both before and after the 6-month stay. Their stool samples were inoculated on chromID™ ESBL, CHROMID® Carba and CHROMID® OXA-48 media (bioMérieux, Marcy l’Etoile, France). Subsequently, bacterial isolates (1–4 per sample) were screened for ESBL and AmpC production, using the ESBL double-disk synergy test (including cefepime disk) [16], followed by PCRs for the detection of various ESBL or AmpC genes [17]. Carbapenemase activity was assessed by the CIM assay [18]. Species were identified using Vitek 2 (bioMérieux).

All of the 90 ESBL/AmpC-producing isolates (*E. coli* and *Klebsiella pneumoniae*) were subjected to short-read whole-genome sequencing (WGS) by Illumina HiSeq platform (Illumina, San Diego, CA, USA), with reads assembled as previously reported [19]. *E. coli* isolates were phylogrouped [20] using the Clermont Typer web interface at CATIBioMed (http://clermontyping.iame-research.center/). Multi-locus sequence typing (MLST) of *E. coli* [21] and *K. pneumoniae* [22] was performed using mlst (https://github.com/tseemann/mlst) and BIGSdb [23], respectively. New *E. coli* sequence types (STs) were assigned at https://enterobase.warwick.ac.uk/species/index/ecoli. The single-nucleotide polymorphism (SNP)-based analysis was done for *E. coli* with BioNumerics v.7.6.3 (Applied Maths NV, Sint-Martens-Latem, Belgium), using selected sample isolates as references. Resistomes were revealed with ResFinder v. 4. 1 (https://cge.cbs.dtu.dk/services/ResFinder/), with 100% coverage and > 98% identity criteria. Susceptibility testing was performed using the in-house broth microdilution (BMD) assay and BMD commercial Micronaut MDR MRGN plates (Bruker Daltonics, Bremen, Germany). The results were interpreted based on EUCAST (http://eucast.org) or CLSI (doxycycline; http://clsi.org) breakpoints.

## Results

Of the 100 soldiers screened, 17 were colonized with ESBL-Ec (14 CTX-M-15) in June 2019, before leaving for Afghanistan (Table 1). At the end of the mission in November, 73 soldiers tested positive, comprising 12/17 carriers at the departure. The November organisms contained 70 ESBL-Ec (66 CTX-M-15, including five with DHA-1 AmpC), one AmpC-Ec (CMY-42), and two ESBL-producing *K. pneumoniae* (CTX-M-15). All isolates tested carbapenemase-negative.

**Table 1:** Frequency and types of organisms isolated from the intestinal colonization of 100 Polish soldiers of the contingent in Afghanistan, June–November 2019

	June *n* (%)	November *n* (%)
Soldiers, MDR *Enterobacterales* carriers	17 (17.0)	70 + 3^a^ (70.0)
ESBL	17 (17.0)	69 + 3^a^ (69.0)
AmpC		1 (1.0)
β-lactamase producers	17	70 + 3^a^
ESBL-*Enterobacterales*	17 (100.0)	69 + 3^a^ (98.6)
AmpC-*Enterobacterales*		1 (1.4)
ESBL-Ec	17 (100.0)	67 + 3^a^ (95.7)
*E. coli* CTX-M-1 gr	15 (88.2)	66 + 3^a^ (94.3)
*E. coli* CTX-M-15	14 (82.4)	58 + 3^a^ (82.9)
*E. coli* CTX-M-15 + DHA-1		5 (7.1)
*E. coli* CTX-M-1	1 (5.9)	
*E. coli* CTX-M-231		3 (4.3)
*K. pneumoniae* CTX-M-15		2 (2.9)
*E. coli* CTX-M-9 gr (CTX-M-27)	2 (11.8)	1 (1.4)
*E. coli* CMY-2-like (CMY-42)		1 (1.4)

^a^3/100 tested soldiers had the same or almost the same CTX-M-15-producing isolates in June and November; these were not included in the final analysis for November

All but one of the 17 June ESBL-Ec isolates belonged to the commensal phylogroups A (*n* = 12) or B1 (*n* = 4) and represented nine STs (Table 2). A cluster of six phylogroup A CTX-M-15-producing ST515 isolates (0-2 SNPs between each other) was notable, like two indistinguishable B1 ST156 isolates with CTX-M-27. Of the 12 soldiers colonized both in June and November, three had the same or almost the same organism, suggesting the remaining 70 November isolates, mostly ESBL-Ec (*n* = 67; 95.7%), to have been acquired possibly in Afghanistan. A half of the ESBL/AmpC-Ec (*n* = 36; 51.4%) represented the more pathogenic phylogroups D (*n* = 27) and B2 (*n* = 9), and 38 STs overall, with more numerous ST10 (phylogroup A), ST131 (B2) and ST394 (D) (*n* = 8; 11.4% each), and ST69 (D) (*n* = 7; 10%). Only four pairs of November ESBL-Ec (including ST69 and ST394) and the two *K. pneumoniae* isolates were closely related to each other within the pairs (0–13 SNPs). In three other ESBL-Ec pairs (ST10, ST69 and ST131), the genetic relatedness was clear (39–56 SNPs), though not necessarily indicative of direct epidemiological links.

**Table 2:** Phylogroups and STs of 87 ESBL-Ec

Phylogroup	June (*n* = 17)			November (*n* = 70)		
	*n*	(%)	ST (*n*)	*n*	(%)	ST (*n*)
A	12	(70.6)	ST515 (6), ST10 (2), ST43 (2), ST34 (1), ST746 (1)	23	(32.9)	ST10 (8), ST226 (2), ST6438 (2), ST14267 (2), ST34 (1), ST46 (1), ST165 (1), ST227 (1), ST450 (1), ST515 (1), ST746 (1), ST757 (1), ST8676 (1)
B1	4	(23.5)	ST156 (2), ST155 (1), ST1795 (1)	10	(14.3)	ST29 (1), ST99 (1), ST156 (1), ST162 (1), ST336 (1), ST616 (1), ST1136 (1), ST12313 (1), ST11978 (1), ST12637 (1)
B2				9	(12.9)	ST131 (8), ST636 (1)
D	1	(5.9)	ST405 (1)	27	(38.6)	ST394 (8), ST69 (7), ST349 (2), ST405 (2), ST14268 (2), ST38 (1), ST70 (1), ST362 (1), ST2076 (1), ST2914 (1), ST6326 (1)
Clade I				1	(1.4)	ST3042 (1)

The ESBL-Ec strains (June plus November) were characterized by β-lactam susceptibility patterns typical for ESBL producers (Table 3), with almost 100% resistance and/or “susceptibility increased exposure” to penicillins, cephalosporins, and aztreonam. Otherwise, all isolates were susceptible in vitro to piperacillin-tazobactam, ceftolozane-tazobactam, ceftazidime-avibactam, and carbapenems. Regarding non-β-lactams, broader resistance/“susceptibility increased exposure” was observed for fluoroquinolones, doxycycline, trimethoprim, and co-trimoxazole. Aminoglycosides, tigecycline, fosfomycin, nitrofurantoin, and colistin were active against most of the isolates.

**Table 3.** Antimicrobial susceptibility of the 87 ESBL-Ec isolated from soldiers in June and November

Antimicrobials^a,b^	R *n* (%)	I^c^ *n* (%)	S *n* (%)
AMX	87 (100.0)		
AMC	19 (21.8)		68 (78.2)
SAM	40 (46.0)		47 (54.0)
PIP	87 (100.0)		
TZP			87 (100.0)
TEM			87 (100.0)
CXM	87 (100.0)		
CTX^c^	84 (96.6)	2 (2.3)	1 (1.1)
CAZ	40 (46.0)	46 (52.9)	1 (1.1)
CZA			87 (100.0)
FEP	86 (98.9)	1 (1.1)	
CTA			87 (100.0)
ATM	82 (94.3)	4 (4.6)	1 (1.1)
ETP			87 (100.0)
IPM			87 (100.0)
MEM^d^			87 (100.0)
AMK	3 (3.4)		84 (96.6)
GEN	9 (10.3)		78 (89.7)
TOB	11 (12.6)		76 (87.4)
CIP^c^	32 (36.8)	36 (41.4)	19 (21.8)
LEV	30 (34.5)	5 (5.7)	52 (59.8)
DOX	39 (44.8)	15 (17.2)	33 (37.9)
TGC	7 (8.0)		80 (92.0)
TRM	75 (86.2)		12 (13.8)
SXT	70 (80.5)	1 (1.1)	16 (18.4)
FUR			87 (100.0)
FOS^e^			87 (100.0)
CMP	10 (11.5)		77 (88.5)
COL	1 (1.1)		86 (98.9)

^a^Abbreviations: *AMX* amoxicillin, *AMC* amoxicillin/clavulanic acid, *SAM* ampicillin/sulbactam, *PIP* piperacillin, *TZP* piperacillin/tazobactam, *TEM* temocillin, *CXM* cefuroxime, *CTX* cefotaxime, *CAZ* ceftazidime, *CZA* ceftazidime/avibactam, *FEP* cefepime, *CTA* ceftolozane/tazobactam, *ATM* aztreonam, *ETP* ertapenem, *IPM* imipenem, *MEM* meropenem, *AMK* amikacin, *GEN* gentamicin, *TOB* tobramycin, *CIP* ciprofloxacin, *LEV* levofloxacin, *DOX* doxycycline, *TGC* tigecycline, *TRM* trimethoprim, *SXT* trimethoprim/sulfamethoxazole, *FUR* nitrofurantoin, *FOS* fosfomycin, *CMP* chloramphenicol, *COL* colistin

^b^The in-house BMD assay included: AMX, AMC, SAM, TEM, CXM, FEP, ATM, ETP, GEN, TOB, DOX, TRM, and FUR. The Micronaut MDR MRGN assay contained PIP, TZP, CTX, CAZ, CZA, CTA, IPM, MEM, AMK, CIP, LEV, TGC, SXT, FOS, CMP, and COL

^c^Susceptible increased exposure

^d^CTX, MEM, CIP–MIC interpretation for indications other than meningitis

^e^FOS: interpretation for the iv formulation

The resistome analysis of the ESBL-Ec strains revealed various mobile AMR genes (Table 4), which with specific chromosomal mutations corresponded well to the susceptibility patterns. Along with genes of β-lactamases mentioned above, multiple quinolone resistance determinants were common, including combinations of mutations in the chromosomal *gyrA* and/or *parC/E* genes, and acquired *qnrB/S*-like genes. Doxycycline, trimethoprim, and sulfamethoxazole resistance correlated with *tet*(A), *dfr*-, and *sul*-like genes, respectively. The isolates carried various genes of aminoglycoside-modifying enzymes; however, those conferring resistance to clinically-relevant drugs, namely, amikacin, gentamicin, and tobramycin (e.g., *aac(3)-IId*; *aac(6')-Ib-cr*), were rare.

**Table 4:** Acquired AMR genes and fluoroquinolone resistance mutations identified in ESBL-Ec strains isolated from soldiers in June and November

Combinations of resistance determinants	June, *n* = 17 *n* (%)	November, *n* = 70 *n* (%)	Total, *n* (%)
β-lactams	17 (100.0)	70 (100.0)	87 (100.0)
*bla*_CTX-M-15_ & *bla*_TEM-1_ / *bla*_CTX-M-15_ & *bla*_TEM-35_	11 (64.7)	32 (45.7)/2 (2.9)	45 (51.7)
*bla*_CTX-M-15_ / *bla*_CTX-M-1_	2 (11.8)/1 (5.9)	22 (31.4)	25 (28.7)
*bla*_CTX-M-15_ & *bla*_DHA-1_ / +*bla*_TEM-1_		4 (5.7)/1 (1.4)	5 (5.7)
*bla*_CTX-M-15_ & *bla*_OXA-1_ / +*bla*_TEM-1_	1 (5.9)	1 (1.4)/4 (5.7)	6 (6.9)
*bla*_CTX-M-27_		1 (1.4)	1 (1.1)
*bla*_CTX-M-27_ & *bla*_TEM-1_	2 (11.8)		2 (2.3)
*bla*_CTX-M-231_ & *bla*_TEM-1_		3 (4.3)	3 (3.4)
Aminoglycosides	14 (82.4)	51 (72.9)	65 (74.7)
*aph*(6)-Id & *aph*(3″)-Ib & *aac*(3)-IId & *aad*A5 or *aad*A2		4 (5.7)	4 (4.6)
*aph*(6)-Id & *aph*(3″)-Ib & *aad*A5 or *aad*A1 or *aad*A2	3 (17.6)	9 (12.9)	12 (13.8)
*aph*(6)-Id & *aph*(3″)-Ib & *sat*2 / *sat*2		3 (4.3)/2 (2.9)	5 (5.7)
*aph*(6)-Id & *aph*(3″)-Ib	8 (47.1)	12 (17.1)	20 (23.0)
*aad*A5/*aad*A1 or *aad*A2	2 (11.8)/1 (5.9)	11 (15.7)/10 (14.3)	24 (27.6)
Aminoglycosides and quinolones	1 (5.9)	6 (8.6)	7 (8.0)
*aac*(6′)-Ib-cr (D181Y) & *aad*A5 & *aph*(6)-Id & *aph*(3″)-Ib & *aac*(3)-IId		2 (2.9)	2 (2.3)
*aac*(6′)-Ib-cr (D181Y) & *aad*A5 & *aph*(6)-Id & *aph*(3″)-Ib & *aac*(3)-IIe	1 (5.9)	1 (1.4)	2 (2.3)
*aac*(6′)-Ib-cr (D181Y) & *aad*A5 / +*aac*(3)-IIe		2 (2.9)/1 (1.4)	3 (3.4)
Quinolones	14 (82.4)	48 (68.6)	62 (71.3)
*qnr*S1/*qnr*S1 & *qnr*B4	14 (82.4)/0	41 (58.6)/5 (7.1)	60 (69.0)
*qep*A4		2 (2.9)	2 (2.3)
Trimethoprim/sulfonamides	15 (88.2)	64 (91.4)	79 (90.8)
*dfr*A & s*ul*	15 (88.2)	56 (80.0)	71 (81.6)
*dfr*A/*sul*		3 (4.3)/5 (7.1)	8 (9.2)
Tetracyclines	15 (88.2)	43 (61.4)	58 (66.7)
*tet*(A)/*tet*(B)	12 (70.6)/3 (17.6)	26 (37.1)/16 (22.9)	57 (65.5)
*tet*(A) & *tet*(B)		1 (1.4)	1 (1.1)
Other	6 (35.3)	52 (74.3)	58 (66.7)
*mph*(A)	5 (29.4)	41 (58.6)	46 (52.9)
*erm*(B)/*cat*A1		6 (8.6)/4 (5.7)	10 (11.5)
*cml*A1	1 (5.9)		1 (1.1)
*ere*(A)		1 (1.4)	1 (1.1)
*gyrA* and/or *parC/E* mutations	5 (29.4)	33 (47.1)	38 (43.7)
*gyrA(S83A)*	1 (5.9)	1 (1.4)	2 (2.3)
*gyrA(S83L)/gyrA(S83L) & parC(S80I)*		13 (18.6)/2 (2.9)	15 (17.2)
*gyrA(S83L) & gyrA(D87N) & parC(S80I* or *E84K) & parE(L416F)*	4 (23.5)	1 (1.4)	5 (5.7)
*gyrA(S83L) & gyrA(D87N) & parC(S80I) & parE(I464F)*		1 (1.4)	1 (1.1)
*gyrA(S83L) & gyrA(D87N) & parC(S80I) & parC(E84V) & parE(I529L)*		7 (10.0)	7 (8.0)
*gyrA(S83L) & gyrA(D87N) & parC(S80I) & parE(S458A)/+parE(S458T)*		3 (4.3)/1 (1.4)	4 (4.6)
*gyrA(S83L) & gyrA(D87N) & parC(S80I) & parE(S458T)*		1 (1.4)	1 (1.1)
*gyrA(S83L) & parE(I355T* or *I529L)*		2 (2.9)	2 (2.3)
*gyrA(S83V) & parC(p.S80I)*		1 (1.4)	1 (1.1)

## Discussion

Our analysis revealed the 70% rate of acquisition of ESBL/AmpC *Enterobacterales* by a group of Polish soldiers stationing in Afghanistan in 2019. Despite some methodological differences between the studies, this high percentage doubled the 34.5% reported for French soldiers in Afghanistan in 2011 [5], but was similar to the 75% rate of ESBL-Ec acquisition by Dutch citizens travelling in 2012–2013 to the South Asia region, comprising the Afghanistan territory [2]. The notable 17% carriage rate at the departure is difficult to comment because the data on the ESBL-Ec colonization in Polish community has been scarce. A study performed in 2015–2017 in several North European countries included 86 individuals (e.g., primary care patients) from Poland, 8% of whom were colonized [24], comparably to Swedish citizens (6.6%) in the same analysis, or to German population (6.3%) in another work [25]. The notably higher rate in the soldiers might have been due to their clustering within units, including increased risk of common exposure to contaminated food or environment. Only 3/17 “pre-colonized” soldiers came back with the same ESBL-Ec from Afghanistan, nine had other organisms, and five were negative, which corresponded well to recent reports on high dynamics of the ESBL-Ec carriage in travelers to the “ESBL broad- dissemination” regions [26, 27]. Moreover, the only three cases of longer-term ESBL-Ec persistence were concordant with the data on rather short duration of the travel-acquired colonization, with a median of 1–3 months [26, 28, 29].

The 90 ESBL/AmpC organisms from 78 individuals were sequenced. The predominance of ESBL-Ec (~ 96.6%), and the high prevalence of the CTX-M-15 enzyme (~ 94.3%) were congruent with multiple data sets on the general community and, especially, travelers to the “ESBL broad-dissemination” areas [2, 5, 24, 25, 27, 29–31]. Our study showed several cases of the occurrence of the same organism in different soldiers, including the cluster of six *E. coli* ST515 CTX-M-15 at the departure, and five pairs of *E. coli* or *K. pneumoniae* CTX-M-15 at return. These evidenced either transmission of bacteria between the individuals or their acquisition from a common contaminated source. However, the overall high clonal diversity of the organisms indicated their vast majority to have been acquired independently by the participants of the military contingent.

The ESBL/AmpC-Ec diversity was demonstrated by classification of the 17 departure and 70 return isolates into nine and 38 STs, respectively, and by limited or no relatedness between most of the isolates of the same ST. The more frequent STs among the return isolates included the ubiquitous commensal ST10 and common pathogenic ST69 and ST131 lineages [14, 32]. However, the ~11% contribution of ST131 was much lower than in community-acquired and healthcare-associated ESBL-Ec infections [33, 34], as well as carriage in hospitalized patients [17]. Studies on ESBL-Ec colonizing travelers to the “ESBL broad-dissemination” regions usually showed their clonal diversity and unique ST distributions, with limited ST131 incidence [27, 30, 35, 36].

Military contingents in some regions of the world have been at increased risk of colonization with AMR microorganisms, especially ESBL-Ec. Even though all of the carriers identified in our study were healthy individuals, they might also transmit these to vulnerable subjects in their habitats after return, creating so a significant epidemiological threat.

## Data Availability

The datasets generated and/or analyzed during the current study are available in the GenBank BioProject PRJNA993595 under the accession (JAUKWR000000000-JAULAH000000000).
